# Proteomic Remodeling in the Failing Left Ventricle Adapting to Dyssynchrony

**DOI:** 10.1002/prca.70052

**Published:** 2026-08-07

**Authors:** Karin Ljung, Nitha Aima Muntu, Ulrika Reistam, Jonathan A. Kirk, Marcus Ståhlberg

**Affiliations:** ^1^ Department of Medicine Karolinska Institutet and ME Cardiology Heart and Vascular Centre Karolinska University Hospital Solna Stockholm Sweden; ^2^ Department of Cell and Molecular Physiology Loyola University Chicago Stritch School of Medicine Chicago Illinois USA

**Keywords:** cardiac remodeling, CRT, dyssynchrony, heart failure, resynchronization

## Abstract

**Purpose:**

In heart failure, dyssynchrony is associated with accelerated cardiac remodeling and a worse prognosis. Both restored with resynchronization. We have previously developed a mouse model of dyssynchrony and resynchronization and here assess changes in protein expression within that model.

**Experimental design:**

Mice were subjected to ischemia/reperfusion followed by pacemaker implantation. Three groups were defined: (i) sinus rhythm for 4 weeks—synchronous heart failure (SynHF), (ii) right ventricular pacing (RVP) for 4 weeks—dyssynchronous heart failure (DysHF), and (iii) RVP for 2 weeks followed by 2 weeks of sinus rhythm—resynchronized heart failure (ResynHF). Heart tissue was evaluated for protein content with mass spectrometry.

**Results:**

A total of 3324 proteins were detected. The abundance of 589 proteins differed between DysHF and SynHF and 253 between DysHF and ResynHF. The changed proteins in the comparisons to DysHF overlapped to a great extent. Several of these proteins were connected to calcium handling or made up part of the sarcomere.

**Conclusion:**

Adding dyssynchrony to ischemic heart failure resulted in protein dysregulation, which was partly reversed by resynchronization. The dysregulation was characterized by a change in proteins related to contractility, which may be part of the positive inotropic effect and reverse remodeling observed with resynchronization.

**Abbreviations**
ANP: Atrial natriuretic peptide
Anx6: Annexin A6
Col4a3: Collagen IV alpha 3
DysHF: Dyssynchronous heart failure
ECM: Extracellular matrix
FDR: False discovery rate
FS: Fractional shortening
Hspg2: Perlecan
Ighg3: Immunoglobulin heavy constant gamma 3
KEGG: Kyoto Encyclopedia of Genes and Genomes
Lcmt1: Leucine carboxyl methyltransferase 1
LVESD: Left ventricular end systolic diameter
Myl7: Myosin regulatory light chain 2
PKC: Protein kinase C
Pln: Cardiac phospholamban
Ppp1r12c: Protein phosphatase 1 regulatory subunit 12C
ResynHF: Resynchronized heart failure
RVP: Right ventricular pacing
Serca2a: Sarco/endoplasmic reticulum calciumadenosine triphosphatase 2a
SynHF: Synchronous heart failure
Ttn: Titin

## 1 Introduction

In heart failure with reduced ejection fraction, dyssynchrony, typically caused by left bundle branch block, is associated with accelerated left ventricular remodeling and a worse prognosis [1]. Cardiac resynchronization therapy, where pacing of the left and right ventricles or conduction system pacing restores synchrony, reverses this remodeling and reduces mortality [2]. The biological processes behind left bundle branch block‐induced remodeling and its reversal with resynchronization are not completely understood. Findings from a canine model of dyssynchronous heart failure (DysHF) as well as small studies with human cardiac tissue indicate that resynchronization reduces cardiac fibrosis and hypertrophy induced by dyssynchrony [3, 4, 5]. Changes in protein expression have been described in the canine model concerning mitochondrial proteins [6, 7], phospho‐proteomics [8, 9], and recently in tissue samples from the left ventricle [10]. The results point to mitochondrial dysfunction, increased fibrosis, and an increased phosphorylation level with dyssynchrony, all of which may be reversed with resynchronization. However, none of these tissue sources are from model systems where genetic tools can be used to study the mechanistic role of these pathways or proteins in these pathways.

**Statement of Clinical Relevance**

Heart failure with reduced ejection fraction affects 1%–2% of the population, and about 30% of these patients display signs of electrical dyssynchrony on ECG, typically left bundle branch block. Dyssynchrony accelerates cardiac remodeling and increases mortality. Resynchronization will reverse remodeling and reduce mortality and may be achieved with a pacemaker stimulating the right and left ventricles, cardiac resynchronization therapy. The mechanism behind the salutary effect of resynchronization is largely unknown. However, it seems to differ from the neuroendocrine blockade achieved with current medical therapy, since resynchronization has a positive inotrope effect, with an acute increase in contractility. By evaluating differences within the proteome related to dyssynchrony and resynchronization, new mechanisms may be indicated, which could reveal new pharmacologic targets. Novel pharmacological interventions in heart failure are warranted since this disease is common and associated with very low quality of life and excess mortality despite the advances of recent years. In this study, a mouse pacemaker model of dyssynchrony and resynchronization, with the possibility for genetic manipulation, is used to evaluate changes in protein expression.

We have developed a custom‐made pacemaker for mice, where right ventricular pacing (RVP) allows us to study dyssynchrony and resynchronization in heart failure [11]. Recently, we described changes in mRNA expression related to dyssynchrony [12], but the global dysregulation of the cardiac proteome in DysHF is not known. In the mouse model, protein expression has been assessed with the proximity extension assay Olink [13], pinpointing certain proteins but not the entire proteome. Relating to the observed global mRNA changes of the cardiac muscle, we hypothesize that dyssynchrony also results in a dysregulation at the protein level. Therefore, we aimed to characterize the global cardiac proteome in heart failure with dyssynchrony and after resynchronization. Determining possible changes in the proteome may help gain new knowledge about the biological mechanisms behind the positive effects of resynchronization in heart failure.

## 2 Materials and Methods

**FIGURE 1:**
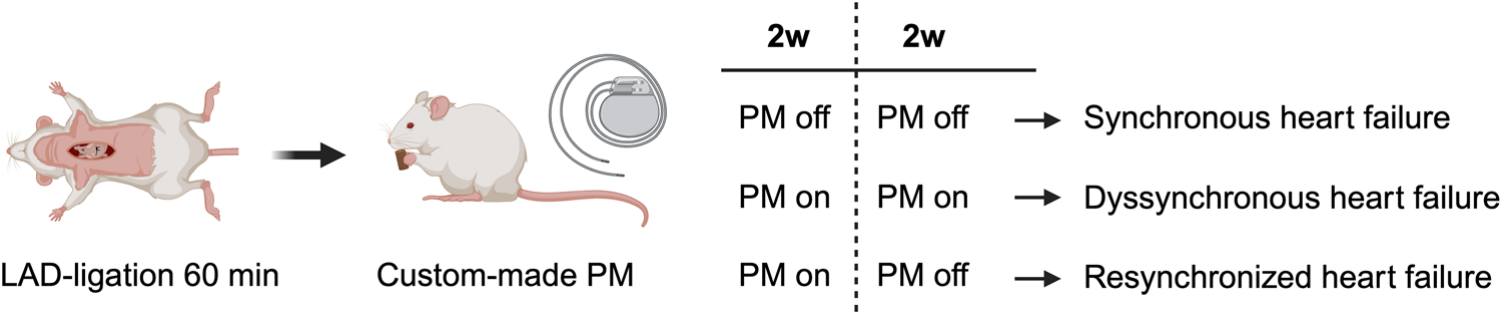
An overview of the mouse model and the different groups. LAD, Left Anterior Descending Artery; PM, pacemaker.

A detailed description of the mouse model of DysHF can be found in the original article [11]. All protocols were reviewed and approved by the Johns Hopkins University Institutional Animal Care and Use Committee (MO13M282). Figure 1 gives an overview of the mouse model of dyssynchrony and resynchronization. Briefly, ischemia/reperfusion for 60 min induced mild to moderate heart failure and mice were subsequently fitted with a custom‐made pacemaker and divided into three groups: sinus rhythm for 4 weeks, synchronous heart failure (SynHF), *n* = 4, RVP for 4 weeks, DysHF, *n* = 5 and RVP for 2 weeks followed by 2 weeks in sinus rhythm, resynchronized heart failure (ResynHF), *n* = 5. RVP for 4 weeks accelerated remodeling, as fractional shortening (FS) was reduced and left ventricular end systolic diameter (LVESD) was significantly increased in DysHF compared to SynHF. FS and LVESD did not change post ischemia/reperfusion in SynHF. In DysHF, FS decreased from 36% (± 6) to 24% (± 6), and LVESD increased from 2.4 mm (± 0.4) to 3.1 mm (± 0.6). Resynchronization reversed remodeling back to the original state, as FS decreased (36.2% ± 2.8–28% ± 1.8) and LVESD increased (2.1 mm ± 0.2–2.8 mm ± 0.1) during the first 2 weeks of RVP but returned to the initial state after 2 weeks without RVP and at the end of the experiment did not differ from SynHF [11].

Mass spectrometry was used to quantify protein levels in heart tissue. The heart tissue was a combination of septum and lateral left ventricular wall in all analyses; tissue from different animals was analyzed separately. Frozen tissues were delicately cut and placed in a glass vial. They were then homogenized at 7500 rpm for 2–3 s pulses in a lysis buffer (9 molar Urea solution) supplemented with phosphatase and protease inhibitors at a 1:100 ratio with 30 µL of 10% Triton X‐100 to lyse and permeabilize the cells. The homogenates were then transferred to Eppendorf tubes, briefly sonicated, and centrifuged at 18,000 g for 10 min. The protein concentration was determined using a Bicinchoninic Acid assay (Thermo Fisher). This was followed by chloroform‐methanol precipitation, resulting in dry protein material that is free of salt and detergent when preparing samples for MS/MS analyses.

Purified peptides, 1.0 ug, were loaded onto a Vanquish Neo UHPLC system (Thermo Fisher) with a heated trap and elute workflow with a c18 PrepMap, 5 mm, 5uM trap column (P/N 160454) in a forward‐flush configuration connected to a 50 cm Easyspray analytical column (P/N ES75500PN) 2uM,100A,75um x 500 mm with 100% Buffer A (0.1% formic acid in water) with flow rate of 0.300 µL and the column oven operating at 35 °C. Peptides were eluted over a 150 min gradient, using 80% acetonitrile 0.1% formic acid (buffer B), going from 4% to 10% over 10 min, to 35% over 100 min, then to 50% over 22 min, then to 99% over 6 min and kept at 99% for 12 min, after which all peptides were eluted. Spectra were acquired with an Orbitrap Eclipse.

Tribrid mass spectrometer (Eclipse) with FAIMS Pro interface (Thermo Fisher Scientific) running Tune 3.5 and Xcalibur 4.5 was used for all acquisition methods, spray voltage set to 1600 V, and ion transfer tube temperature set at 300oC; FAIMS switched between CVs of −45, −55, and −65 V with cycle times of 1.5 s. MS1 spectra were acquired at 120,000 resolutions with a scan range from 375 to 1600 *m/z*, normalized AGC target of 300%, and maximum injection time set to auto, S‐lens RF level set to 30 without source fragmentation and datatype positive and profile; Precursors were filtered using monoisotopic peak determination set to peptide MIPS; included charge states, 2–7 (reject unassigned); dynamic exclusion enabled, with *n* = 1 for 60s exclusion duration at 10 ppm for high and low. DDMS2 scan using isolation mode quadrupole, isolation window (*m/z*): 1.6; activation type set to HCD with 30% collision energy (CE), detector type: ion trap; scan rate: turbo; AGC target: 10,000; maximum injection time: 35 ms, microscans: 1 and data type: centroid.

Raw data and statistical analysis were performed using Proteome Discoverer 2.5 (Thermo Fisher) with Sequest HT search engines. Missing values were also analyzed by Proteome Discoverer 2.5, and imputation was not used. Overall, missing values averaged 10.4% for each sample at the protein level, although for ∼70% of protein IDs there was only 0 or 1 missing value across all samples. The data were searched against the Mus Musculus entries in the UniProt protein sequence database (Mus Musculus, Proteome ID UP000000589). The request search parameters included a precursor mass tolerance of 10 ppm and 0.6 Da for fragments, two missed trypsin cleavages, oxidation (Met) and acetylation (protein N‐term) as variable modifications, and carbamidomethylation (Cys) as a static modification. Percolator PSM validation was used with the following parameters: strict false discovery rate (FDR) of 1%, relaxed FDR of 5%, maximum Δ*C*n of 0.05, and validation based on *q*‐value. Precursor ions quantifier settings were peptides to use: unique + razor; consider protein groups for peptide uniqueness set as true; Precursor abundance based on: Intensity; Normalization based on total peptide amount; scaling mode set as none. Protein abundances were calculated from summed intensity values, and abundance ratios were determined using a class comparison hypothesis test based on a background‐corrected t‐test. To adjust for multiple testing, a false discovery rate (FDR) of 5% was applied after pairwise comparisons. *p* values presented in the paper refer to 5% FDR‐adjusted *p* values. A *p* < 0.05 was considered significant. The David Database [14] was used for pathway analysis.

## 3 Results

**FIGURE 2:**
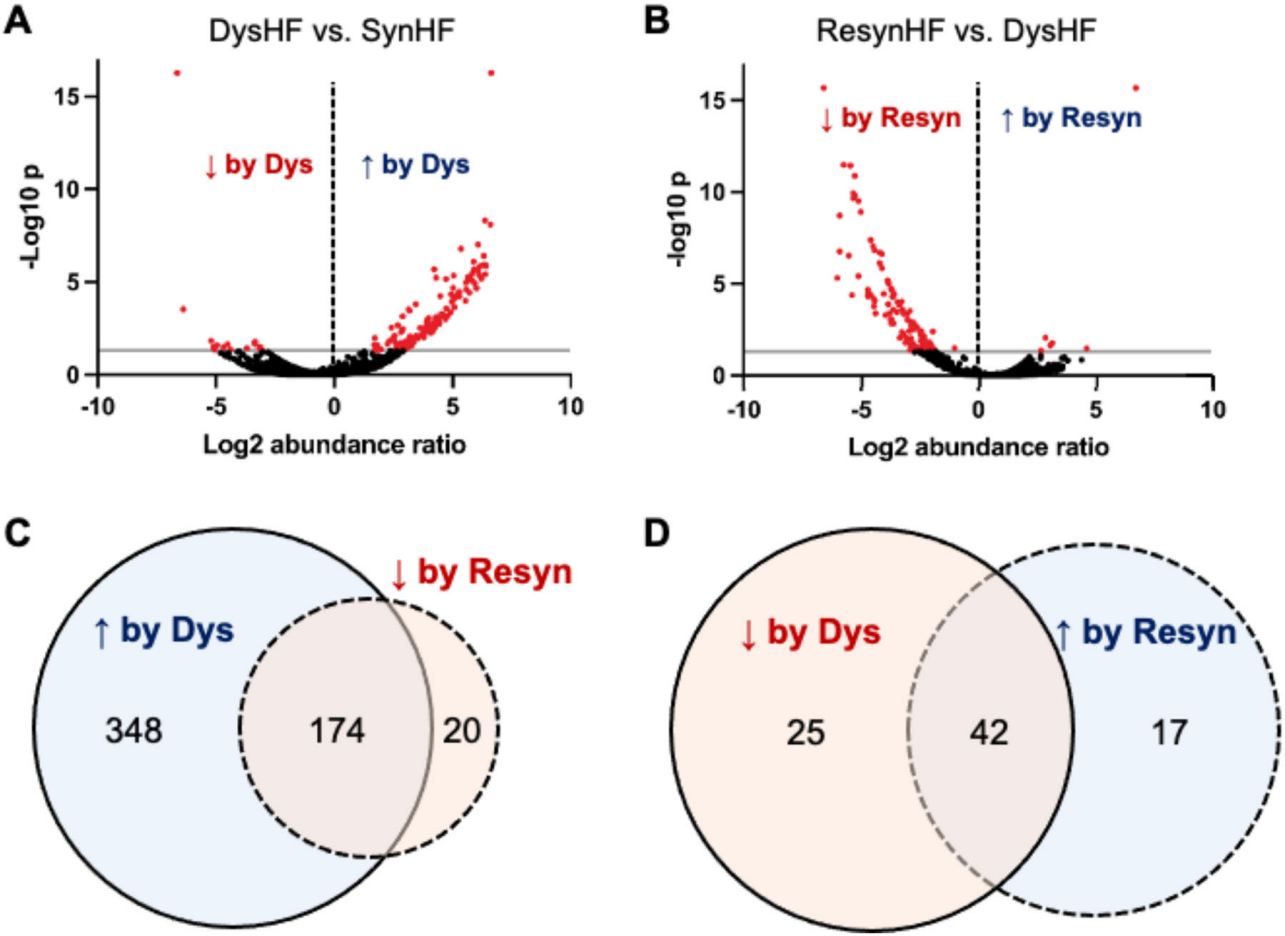
(A) and (B) are volcano plots that give the difference in protein abundance between DysHF (*n* = 5) and SynHF (*n* = 4) or ResynHF (*n* = 5). −Log10 of the 5% FDR‐adjusted *p* value is given on the *y*‐axis and Log2 of the abundance ratio on the *x*‐axis. The dots correspond to one protein, and red dots indicate that the protein abundance significantly changed. Proteome Discoverer 2.5 was used to calculate *p* values and FDR for all comparisons. (C) and (D) are VENN diagrams illustrating the overlap in changed protein abundance between the two comparisons. DysHF, Dyssynchronous heart failure; FDR, False detection rate; ResynHF, Resynchronized heart failure; SynHF, Synchronous heart failure.

Among all three groups, SynHF (*n* = 4), DysHF (*n* = 5), and ResynHF (*n* = 5), we detected a total of 3324 proteins in the combined tissue from septum and left lateral wall, reflecting global left ventricular protein expression. When comparing DysHF to SynHF (the impact of dyssynchrony in the setting of heart failure), we found 589 differentially expressed proteins (18%); 522 were upregulated and 67 downregulated in DysHF (Figure 2A and Table S1). These results indicate that dyssynchrony broadly results in an increase in protein expression. Comparing ResynHF to DysHF (the impact of resynchronization), 253 proteins (8%) were differentially expressed: 194 were downregulated in ResynHF, and 59 were upregulated in DysHF (Figure 2B and Table S2), indicating that resynchronization broadly reduces protein expression (back toward SynHF).

The overlap between these groups shows how effective resynchronization is in reversing the proteomic remodeling associated with dyssynchrony, in the setting of heart failure. Of the 522 proteins upregulated by dyssynchrony, 174 proteins were decreased by resynchronization, reversing one third of the upregulated remodeled proteome (Figure 2C and Table S3). Of the 67 proteins downregulated by dyssynchrony, 42 (63%) of them were increased by resynchronization (Figure 2D and Table S4). Thus, overall, resynchronization reversed 37% (216/589) of the dysregulated proteins in dyssynchrony. Conversely, 85% (216/253) of the proteins altered by resynchronization were reversing changes caused by dyssynchrony. Among the resynchronization‐corrected proteins, we noted several connections to sarcomere function and calcium handling, such as Titin (Ttn), Protein phosphatase 1 regulatory subunit 12C (Ppp1r12c), and cardiac phospholamban (Pln), as well as proteins important to the extracellular matrix like perlecan (Hspg2) and several collagens. Proteins that changed abundance with dyssynchrony and were not corrected by resynchronization included galectin 3, protein kinase C (PKC), and atrial natriuretic peptide (ANP).

When examining the proteins that were exacerbated by resynchronization (i.e., the direction of change was the same for both dyssynchrony and resynchronization), this list was significantly smaller. Zero proteins were increased by both dyssynchrony and resynchronization, and only three proteins were decreased by both dyssynchrony and resynchronization (Table S5): Myosin regulatory light chain 2 (Myl7), Immunoglobulin heavy constant gamma 3 (Ighg3), and leucine carboxyl methyltransferase 1 (Lcmt1). Selected proteins from each group are shown in Table 1.

**TABLE 1:** The names of selected proteins with changed abundance.

	Dys	Resync	Gene	Protein name
Altered by Dyssynchrony	↑	↔	Capzb	Capping protein (Actin filament) muscle Z‐line
	↑	↔	Lgals3	Galectin‐3
	↑	↔	Trim28	Transcription intermediary factor 1‐beta
	↑	↔	Bak1	BCL2‐antagonist/killer 1
	↑	↔	Nppa	Natriuretic peptides A
	↑	↔	Prkcd	Protein kinase C
	↑	↔	Mak	Serine/threonine‐protein kinase MAK
	↑	↔	Bnip3	BCL2/adenovirus E1B 19 kDa protein‐interacting protein 3
	↑	↔	Arpc1b	Actin‐related protein 2/3 complex subunit 1B
	↑	↔	Ablim1	Actin‐binding LIM protein 1
	↑	↔	Prkacb	cAMP‐dependent protein kinase catalytic subunit beta
	↑	↔	Col4a3	Collagen alpha‐3(IV) chain
	↓	↔	Rbp1	Retinol‐binding protein 1
	↓	↔	Aldoc	Fructose‐bisphosphate aldolase C
CRT Corrected	↑	↓	Ttn	Titin
	↑	↓	Col6a3	Collagen, type VI, alpha 3
	↑	↓	Hspa2	Heat shock‐related 70 kDa protein 2
	↑	↓	Pln	Cardiac Pln
	↑	↓	Ppp1r12c	Protein phosphatase 1 regulatory subunit 12C
	↑	↓	Mtfr1l	Mitochondrial fission regulator 1‐like
	↑	↓	Fxyd1	Phospholemman
	↑	↓	Pcp4l1	Purkinje cell protein 4‐like protein 1
	↓	↑	Hspg2	Perlecan
	↓	↑	Mtco1	Cytochrome c oxidase subunit 1
	↓	↑	Mylk	Myosin light chain kinase, smooth muscle
	↓	↑	Acly	ATP‐citrate synthase
CRT Only	↔	↓	Hsd17b11	Estradiol 17‐beta‐dehydrogenase 11
	↔	↑	Ttk	Dual specificity protein kinase TTK
	↔	↑	Anxa6	Annexin A6
	↔	↑	Plcb4	Phospholipase C, beta 4 (fragment)
Same	↓	↓	Ighg3	Immunoglobulin heavy constant gamma 3 (fragment)
	↓	↓	Lcmt1	Leucine carboxyl methyltransferase 1
	↓	↓	Myl7	Myosin regulatory light chain 2, atrial isoform

The arrows to the right indicate whether they were up‐ or down‐regulated or not significantly (ns) changed in the two comparisons. Abbreviations: SHF, Synchronous heart failure; DHF, Dyssynchronous heart failure; RHF, Resynchronized heart failure.

**FIGURE 3:**
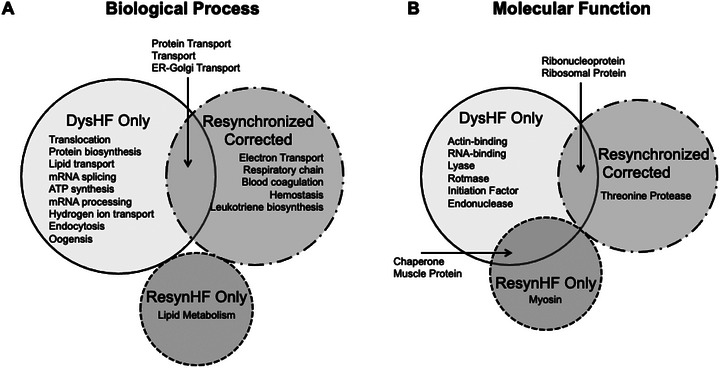
(A) The affected pathways for biological processes. (B) The affected pathways for molecular function in the comparisons between DysHF (*n* = 5) and SynHF (*n* = 4) or ResynHF (*n* = 5). DysHF, Dyssynchronous heart failure; ResynHF, Resynchronized heart failure; SynHF, Synchronous heart failure.

We then performed pathway analysis on three categories of proteins: those dysregulated (up or down) by dyssynchrony that were not corrected by resynchronization (370 proteins, DysHF only), those dysregulated by dyssynchrony that were corrected by resynchronization (216 proteins, ResynHF corrected), and those that were altered by resynchronization but were not dysregulated by dyssynchrony (37 proteins, ResynHF only). We performed both Kyoto Encyclopedia of Genes and Genomes (KEGG) molecular function and biological pathway analysis (Table S6). It is possible that terms could overlap between multiple groups (some elements of that pathway are corrected, and others are not). For the biological pathway analysis, the pathways that only appeared in the “DysHF only” group included: translocation, protein biosynthesis, and lipid transport. Pathways that only appeared in the “resynchronization corrected” group included: electron transport, respiratory chain, and blood coagulation. Only one term appeared only in the “ResynHF only” group: lipid metabolism (Figure 3A).

For the KEGG molecular function analysis, the terms that appeared only in the “DysHF only” group included: actin‐binding, RNA‐binding, and lyase. In the “resynchronization corrected” group, only threonine protease was unique, and in “ResynHF only” the term myosin was unique (Figure 3B).

## 4 Discussion

Dyssynchrony added to a moderate ischemic heart failure model induced a pronounced change in the proteome compared to synchronous heart failure. The marked overlap in up‐ or down‐regulation of proteins in both comparisons to DysHF indicates that proteomic changes in DysHF are reversed with resynchronization, which agrees well with the remodeling [11] and the changes in mRNA expression previously described in the same model [12]. Pathway analysis revealed that for proteins corrected by resynchronization, not corrected by resynchronization, and those only affected by resynchronization (no difference between SynHF and DysHF), specific biological pathways were enriched. These results indicate that there are specific pathways that resynchronization is able to correct, and pathways it is unable to correct. Interestingly, there are also pathways which resynchronization modifies, but that were not modified by dyssynchrony to begin with.

The positive inotrope effect of CRT has been connected to changes in sarcomere proteins, mainly changed phosphorylation, and increased calcium sensitivity [3]. Among the proteins corrected by resynchronization, Pln may be relevant, since mutations in humans cause dilated cardiomyopathy and arrhythmogenic cardiomyopathy, likely through its interaction with sarco/endoplasmic reticulum calcium adenosine triphosphatase 2a (Serca2a) [15]. Pln was assessed by Western blot in an earlier study with dyssynchrony and resynchronization in canines [16]. This study focused on electrophysiology and found a downregulation of Pln in dyssynchrony and resynchronization compared to controls. Our finding, where resynchronization restores Pln to a lower level compared to dyssynchrony, could indicate decreased inhibition of Serca2a. However, the biologic activity of Pln is highly related to phosphorylation status [15], which we did not study here, but has been studied earlier in the canine model without alterations [9]. Our findings indicate a role for Pln in resynchronization therapy and support the importance of calcium handling.

Dyssynchrony and resynchronization also affected several proteins making up the sarcomere; titin was corrected by resynchronization back to SynHF levels, but another sarcomeric protein, myosin regulatory light chain 2a (Myl7), which was downregulated in DysHF compared to SynHF, was then further downregulated in ResynHF compared to DysHF. Meaning that resynchronization here causes an even more profound dysregulation compared to SynHF, even though the phenotype has returned. This finding indicates that resynchronization may not be simply a reversal back to synchrony, but perhaps new pathways are activated. We found three proteins fitting this description (Tables 1 and S5), where Myl7, which regulates calcium binding to myosin and where deficiency was recently connected to cardiac hypertrophy in mice [17], is likely most relevant. The Myl7 activity depends on its phosphorylation status, where phosphorylation promotes the myosin head interacting with the thin filaments [18]. Myosin light chain kinase 3 (Mylk3) phosphorylates the myosin regulatory light chain in the heart [19] and did not change expression in our material. The dephosphorylation is achieved through protein phosphatase 1 and facilitated by protein phosphatase 1 regulatory subunit 12C (Ppp1r12c) [20]. Ppp1r12c is among the proteins that are upregulated in DysHF and corrected by resynchronization, which points to the fact that both the concentration and phosphorylation state of Myl7 change in ResynHF. In atrial fibrillation, increased levels of Ppp1r12c decrease Myl7 phosphorylation in mice, and in patients with atrial fibrillation, an increase in Ppp1r12c was connected to reduced atrial contractility [20]. These changes in Myl7 expression and, possibly, phosphorylation status could be part of the mechanism behind the earlier findings from this mouse model of increased contractility with unchanged calcium flux in ResynHF [11] as well as the findings from the canine model of increased calcium sensitivity [3], and need to be further explored.

The proteins that were ResynHF exclusive, only changed after resynchronization, not due to dyssynchrony, deserve extra attention. These were fewer and engaged pathways related to myosin, muscle proteins, and lipid metabolism, which again relates to energy consumption and contractility. Among the proteins only changed by resynchronization, annexin A6 (Anx6), a protein involved in plasma membrane repair after injury [21], increased. An earlier study that utilized the canine model of tachypacing and focused on mitochondrial proteins found the opposite pattern, with an increase in heart failure and a decrease with CRT [7]. However, Gong et al. isolated and analyzed only mitochondria, while we assessed entire cardiac tissue, pointing to an increase located in parts of the cell other than the mitochondria. Earlier studies have implicated Anx6 in heart failure pathology, but with diverging results [22, 23], and since the studies were done more than 20 years ago, the techniques differed a lot from what is used today. The fact that Anx6 appears in several different studies and models of heart failure indicates that it may play a part in pathogenesis, but further studies are needed to gain a full understanding.

Changes in ECM components in DysHF and cardiac remodeling have been described before in terms of increased fibrosis [4, 24, 25]. We found that dyssynchrony resulted in an upregulation of several collagen chains, in agreement with earlier studies. The changes in two basement membrane proteins with dyssynchrony, collagen alpha‐3(IV) chain (Col4a3) and perlecan (Hspg2), where resynchronization could restore Hspg2 constitute a novel finding. Col4a3 increased, and perlecan decreased with dyssynchrony, which indicates a skewing of the composition of the basement membranes. Perlecan is important for vasculogenesis and, among other things, regulates smooth muscle cell growth in the vessel wall [26]. A perlecan deficiency in the basement membranes in DysHF could cause dysregulated and increased smooth muscle cell growth in the vessel wall, which in turn could be part of the endothelial dysfunction found in DysHF [27].

Earlier studies of DysHF have focused on selected parts of the proteome [7, 9], but recently a study of the full cardiac tissue proteome from the canine model of dyssynchrony and resynchronization was published [10]. Mass spectrometry was utilized, but only 15 proteins differed between dyssynchrony and resynchronization, and these did not overlap with our findings. The differences are likely related to both the difference in models and the difference in tissue used for analysis. The canine model is a model of tachypacing, while the mouse model induces heart failure by ischemia, which is then aggravated by RVP, set just above the normal heart rhythm to avoid tachypacing. The tissue used by Wu et al. originated solely from the left ventricle, while we have used a combination of septum and lateral left ventricular wall. We believe that omitting septum from the analysis may lead to the loss of important information and a reduction in the number of proteins changed. Our findings from mRNA sequencing support this [12], indicating that the changes are at least as profound in septum as in the left wall. Echocardiographic changes found in human patients responding to CRT paint the same picture, with large effects on myocardial work in the septum as part of the response to CRT [28], as do earlier findings of hypoperfusion of the septal tissue in canines with dyssynchrony [29].

The only earlier study concerning protein expression in the same mouse model could not show a difference between groups but within the heart muscle [13]. The lack of between‐group differences is likely due to different methods, where the earlier study utilized a proximity extension assay (Olink) to assess the concentration of 92 proteins, while the current study aims to assess the entire proteome using mass spectrometry. Several earlier studies have examined protein expression in the canine model with a focus on mitochondrial proteins [6, 7] or function [30], since resynchronization is the only heart failure treatment with a positive inotrope effect and decreased mortality [2, 31] and early studies showed that the increased inotropy was achieved without increased oxygen consumption [32], which may be connected to changes in the mitochondria. We have not studied mitochondrial proteins separately; however, the pathway analysis indicates that dyssynchrony affects energy production (ATP synthesis), and findings from mRNA sequencing in the same model [12] indicated upregulation of DNA and RNA synthesis. This could reflect changes in mitochondrial processes and multiplication.

Dyssynchrony led to profound changes in the proteome, where known protein biomarkers in heart failure were affected; Galectin 3, ANP, and PKC were all increased with dyssynchrony but not corrected by resynchronization. Galectin 3 has been extensively studied as a biomarker for disease, and increased levels have been connected to a worse prognosis after myocardial infarction and in chronic heart failure [33]. Therefore, an increase in DysHF is expected. ANP is a natriuretic peptide produced by cardiomyocytes and known to increase with heart failure [34] and was, not surprisingly, further increased with dyssynchrony in this model. PKC is known to increase in ischemia and heart failure [35] and, in this model, further increased with the aggravated heart failure in DysHF. The increase of these three proteins in DysHF was expected, but one would also expect a correction by resynchronization, as it corrected the phenotype in ResynHF. However, this was not the case, and in all, 370 of the 589 proteins changed by dyssynchrony were not corrected by resynchronization, even though the heart function returned to SynHF level. A possible explanation is that 2 weeks is too short for the full protein turnover to take place as a result of the biological processes started by resynchronization. That would mean that some of the proteins that do not have a significantly different abundance after resynchronization would have after a few more weeks. This notion is supported by a recent study evaluating the turnover of sarcomeric proteins in adult mice and found the turnover half‐life to be about 7–9 days for Titin [36]. In our model, the mice were euthanized 14 days after resynchronization, meaning that equilibrium in protein turnover after induced changes by resynchronization was not yet reached. DysHF mice were euthanized after 28 days of RVP and would be very close to equilibrium in protein turnover. This implies that some of the changes in protein expression induced by resynchronization were probably not recognized here. In future experiments, several time‐points and a longer time until euthanasia could answer this question.

The small sample size in this study, and hence, the statistical power to detect differences in protein expression between the groups is a limitation. Therefore, we may miss differences between groups, and a larger study, around 15 mice per group, would have reduced this problem. However, due to the complexity of the animal model, a large sample size is challenging. The small sample size in the present study can be justified by the fact that all mice are genetically identical, phenotypically very similar (in terms of age, sex, body size, absence of comorbidities, etc.), and in a rigorously controlled experiment where the only difference between the study groups is pacing mode. An additional risk with the small sample size in this experiment is model overfitting and false positive findings. We applied a statistical method (false discovery rate) to avoid false positive findings but cannot rule out that some proteins differed between the groups by chance. Another limitation is the lack of clinical parameters, except for echocardiography. This limitation is related to the small animal model used, where both the possibility of blood sampling and symptom assessment are restricted.

The tissue‐specific changes in protein expression observed in this study may aid in the understanding of the mechanisms behind the positive effects of resynchronization. That, in turn, could provide us with new targets for pharmacological treatment of heart failure. However, the translational potential of these findings needs confirmation in studies on larger mammals.

Adding dyssynchrony on top of moderate ischemic heart failure results in a significant protein dysregulation. Resynchronization will partly reverse this but also induce new changes, which may constitute clues to the mechanism behind the positive effects of resynchronization. Several of the changed proteins were involved in calcium handling (Pln) and contractility (Ttn, Myl7, Ppp1r12c) where the changes observed agree with the inotrope effect of resynchronization. These findings constitute a foundation for further research into the importance of specific proteins for dyssynchrony‐associated cardiac remodeling using a genetically modifiable rodent model.

## Funding

This work was supported by The Swedish Heart Lung Foundation [20200616 to M.S.] and the National Institute of Health [R01HL136737 to J.A.K.].

## Conflicts of Interest

The authors declare no conflicts of interest.

## Supporting information




**Supporting File 1:** prca70052‐sup‐0001‐TableS1.xlsx.

**Supporting File 2:** prca70052‐sup‐0002‐TableS2.xlsx.

**Supporting File 3:** prca70052‐sup‐0003‐TableS3.xlsx.

**Supporting File 4:** prca70052‐sup‐0004‐TableS4.xlsx.

**Supporting File 5:** prca70052‐sup‐0005‐TableS5.xlsx.

**Supporting File 6:** prca70052‐sup‐0006‐TableS6.xlsx.

## Data Availability

All mass spectrometry data used for the generation of this manuscript have been deposited into the online respository MassIVE (https://massive.ucsd.edu/ProteoSAFe/dataset.jsp?task=bc03972edab84f1cbf1923779b75d728) and is available with the username: MSV000098672_reviewer.
